# Time- and Genotype-Dependent Root-Transcriptomic Responses of Soybean to Combined Soybean Aphid and Soybean Cyst Nematode Infestation

**DOI:** 10.3390/plants15132014

**Published:** 2026-06-29

**Authors:** Surendra Neupane, Adam J. Varenhorst, Madhav P. Nepal

**Affiliations:** 1 Department of Clinical Science, H. Lee Moffitt Cancer Center, Tampa, FL 33612, USA; surendra.neupane@moffitt.org; 2 Department of Agronomy, Horticulture & Plant Science, South Dakota State University, Brookings, SD 57007, USA; adam.varenhorst@sdstate.edu; 3 Department of Biology & Microbiology, South Dakota State University, Brookings, SD 57007, USA

**Keywords:** soybean defense, soybean aphid, soybean cyst nematode, root transcriptomics, aboveground–belowground interactions

## Abstract

The soybean aphid (*Aphis glycines*) and soybean cyst nematode (*Heterodera glycines*) are major aboveground and belowground pests of soybean (*Glycine max*) in the U.S. Midwest, but the molecular basis of their combined effects on soybean defense remains poorly understood. This study examines how soybean genotypes influence demographic and root-transcriptomic responses to single and combined pest infestation. Soybean cyst nematode reproduction increased under combined infestation in the susceptible cultivar but remained unchanged in the resistant cultivar, whereas soybean aphid populations declined when plants were also infested with nematodes. Root RNA-seq revealed strong time-dependent transcriptional responses, with substantially more differentially expressed genes at 30 days post-infestation than at 5 days post-infestation. Co-expression and enrichment analyses showed that early responses were associated with defense signaling, plant–pathogen interaction, and cutin, suberin, and wax biosynthesis, whereas later responses involved redox processes, isoflavonoid biosynthesis, phenylpropanoid metabolism, and one-carbon metabolism. Several differentially expressed soybean genes co-localized with known soybean cyst nematode resistance quantitative trait loci, including genes near the *rhg1* region. Together, these results suggest that soybean genotypes strongly influence soybean aphid–soybean cyst nematode interactions and identify candidate genes and pathways that may contribute to durable resistance against interacting aboveground and belowground pests.

## 1. Introduction

Soybean, *Glycine max* (L.) Merr., is a globally important crop valued for its high-quality protein and oil [1,2]. In the Midwestern United States, the soybean aphid (SBA), *Aphis glycines* Matsumura (Hemiptera: Aphididae), and soybean cyst nematode (SCN), *Heterodera glycines* Ichinohe (Tylenchida: Heteroderidae), are the two most economically significant pests [3,4]. The soybean aphid is an aboveground phloem-feeding herbivore, whereas the SCN is a belowground parasite of soybean roots. Infestations with these pests often co-occur and can further exacerbate yield losses [5,6]. Annual economic losses in the United States without proper management practices are estimated at ~$4 billion for the SBA and $1.3 billion for the SCN [7,8,9,10]. Management strategies primarily rely on host plant resistance (i.e., for SCNs) and insecticidal management (i.e., for SBAs) [11,12,13]. However, the recent discovery of pyrethroid-resistant SBA populations in Iowa, Minnesota, North Dakota, and South Dakota has highlighted the need for the adoption of alternative SBA management tactics, including host plant resistance [13]. Similarly, long-term deployment of SCN resistance has selected for virulent SCN populations capable of overcoming resistance genes (i.e., HG types) [14]. Although host resistance is not widely implemented for SBAs, multiple virulent SBA biotypes have been identified in the U.S.; together, the evolution of virulent SBA biotypes and SCN races threatens the durability of host plant resistance [14,15,16,17]. Thus, genetic insights from greenhouse-based experiments on SBA and SCN interactions are critical for identifying resistance genes and regulatory networks that can support durable resistance to both pests in soybean.

Despite occupying distinct niches, above- and belowground herbivores share a host through systemic tissues and can influence each other [18]. Prior studies have investigated SBA–SCN interactions in soybean using demographic approaches [5,6,19,20,21]. For example, McCarville et al. [5] showed that SCN reproduction increased 5.24-fold in the presence of SBAs and the fungus *Cadophora gregata*, while aphid populations decreased by 26.4% when SCNs and the fungus were present. Later, McCarville et al. (2014) [6] found that SBA feeding improved soybean’s suitability as a host for SCNs, although outcomes varied with cultivar and experiment duration. These demographic studies highlight the complexity of SBA–SCN interactions, but their molecular underpinnings remain unexplored. While both soybean aphids (SBAs) and soybean cyst nematodes (SCNs) interact with a host through different tissues (leaves vs. roots), root transcriptomics was prioritized in this study because the SCN is a root pathogen that establishes specialized feeding sites (syncytia), making roots the primary site of nematode infection and host response.

Transcriptomics with RNA sequencing (RNA-Seq) is a widely used approach for both qualitative and quantitative gene expression profiling, providing detailed insights into transcript abundance and regulatory dynamics [22,23]. In particular, root RNA-seq provides a suitable approach for addressing the current knowledge gap because the SCN directly modifies root tissues, while SBA feeding may induce systemic root responses that alter nematode performance and host defense status [24,25]. In addition, known SCN resistance loci and defense-related soybean genes, including genes associated with *rhg1*, *Rhg4*, lignin deposition, vesicle trafficking, oxidative stress, and secondary metabolism, provide a framework for connecting differential gene expression with candidate mechanisms of durable resistance [26,27,28,29,30].

We hypothesized that combined SBA and SCN infestation would induce time-dependent and genotype-specific root-transcriptomic responses, with resistant and susceptible cultivars differing in defense-related gene expression and pathway activation. To test this hypothesis, we had two main objectives: first, to evaluate genotype-dependent demographic interactions between SBAs and SCNs in resistant and susceptible soybean cultivars, and second, to characterize early and late root-transcriptomic responses to single and combined pest infestation, including shared DEGs, resistant-cultivar candidate genes, enriched pathways, co-expression modules, and QTL-coincident genes.

## 2. Results

### 2.1. Greenhouse Experiment Reveals Asymmetric Aboveground–Belowground Interactions

**Figure 1:**
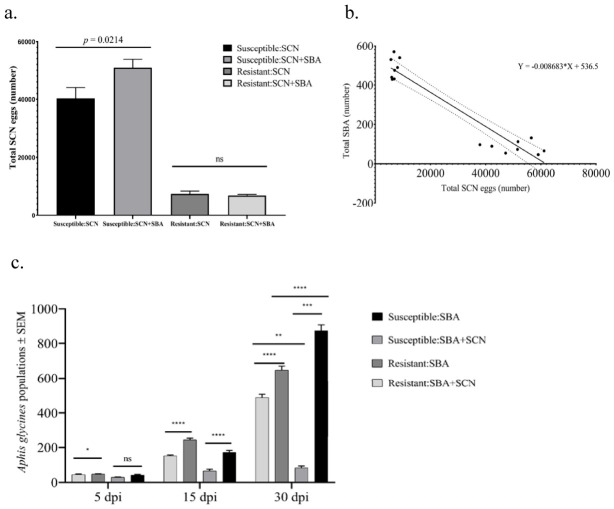
Soybean aphid (SBA) and soybean cyst nematode (SCN) responses in susceptible Williams 82 and resistant MN1806CN soybean. (**a**) SCN egg counts at 30 days post-infestation (dpi) under SCN-only and SCN + SBA treatments. (**b**) Descriptive relationship between total SCN eggs and total SBA abundance at 30 dpi. (**c**) SBA biotype 1 population size at 5, 15, and 30 dpi under SBA-only and SBA + SCN treatments. Bars indicate mean ± SEM. Asterisks indicate significant differences for the indicated post hoc multiple-comparison tests within the relevant cultivar and/or time point: ns, not significant; *, *p* ≤ 0.05; **, *p* ≤ 0.01; ***, *p* ≤ 0.001; ****, *p* ≤ 0.0001.

We quantified SCN reproduction (egg counts at 30 dpi or days post-infestation) and SBA population growth (5, 15, and 30 dpi) across SCN-resistant (MN1806CN) and SCN-susceptible (Williams 82; PI 518671) cultivars under four treatment conditions (control, SBA, SCN, and combined SBA + SCN). SCN egg counts differed among treatments at 30 dpi. In the SCN-resistant cultivar, egg numbers were not significantly different between the SCN-only and SCN + SBA treatments. In contrast, in the susceptible cultivar, SCN egg numbers were significantly higher under combined SCN + SBA infestation than under SCN infestation alone (Figure 1a). Soybean aphid abundance at 30 dpi was lower in plants also infested with SCNs, with a stronger reduction observed in the SCN-susceptible cultivar than in the SCN-resistant cultivar (Figure 1c). Because soybean genotype and pest treatment strongly influenced both SCN reproduction and SBA population growth, the pooled relationship between SCN egg density and SBA abundance was not used as the primary basis for biological interpretation. Together, these results suggest that SBA–SCN interactions depend on soybean genotype, with resistant and susceptible backgrounds showing different aboveground and belowground pest responses. Figure 1b is presented as a descriptive visualization of the relationship between SCN egg counts and SBA abundance at 30 dpi. Because the points combine cultivars and treatments, this pooled relationship should be interpreted cautiously and should not be considered evidence of a treatment-independent biological association.

### 2.2. RNA-Seq Dataset Quality and Overall Structure of the Transcriptome Signal

Experimental design, library construction, sequencing, and primary read processing (including trimming and read-mapping/quantification) were previously described and quality-validated in our *Scientific Data* paper [31]: root RNA-seq generated >1.1 billion reads across 48 libraries (5 and 30 dpi), with high base quality (Phred > 30), a GC content of ~43–45%, >99% reads retained after trimming, and strong alignment performance (mapped, ~73.8–94.3%; uniquely mapped, ~67.1–87.6%). After filtering, 43,122 genes were retained, and variance-stabilized counts supported robust clustering and ordination; PCA indicated time was the dominant driver of expression variation (PC1 = 28%; *p* = 1.16 × 10^−6^), with treatment also contributing (PC2 = 15%; *p* = 2.02 × 10^−8^). The larger number of DEGs at 30 dpi than at 5 dpi (19,032 vs. 4637 DEGs), along with the larger shared response at 30 dpi (1535 vs. 242 shared DEGs), suggests that soybean roots undergo stronger transcriptional reprogramming at the later stage of pest pressure. This likely reflects SCN development, changes in aphid populations, and broader late-stage defense and metabolic responses rather than technical variation, as read quality, mapping performance, sample clustering, and PCA were consistent across the dataset.

### 2.3. Co-Expression Networks Reveal Late ROS-Associated Root Responses

**Figure 2:**
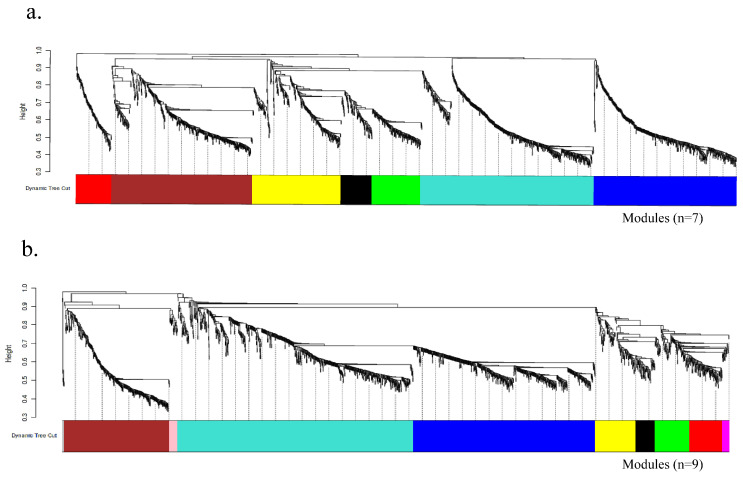
Weighted gene co-expression network analysis (WGCNA) identified a network of 2000 genes divided into seven co-expression modules in (**a**) 5 dpi samples and a network of 1994 genes divided into nine co-expression modules in (**b**) 30 dpi samples. The dendrograms show gene clustering based on co-expression similarity. Each color denotes a distinct co-expression module and does not represent expression magnitude or direction.

To compare early (5 dpi) and late (30 dpi) responses while reducing model complexity, we constructed weighted gene co-expression network analyses (WGCNA) separately for each time point. WGCNA identified seven co-expression modules at 5 dpi (2000-gene network) and nine modules at 30 dpi (1994-gene network) (Figure 2). Hydrogen peroxide and reactive oxygen species metabolic processes were enriched at 30 dpi but not at 5 dpi, suggesting that ROS-associated root responses were more prominent during the later stage of pest pressure.

### 2.4. Large, Time-Dependent Cultivar Contrasts Reveal Shared Core Responses to SCNs, SBAs, and Their Combination

**Figure 3:**
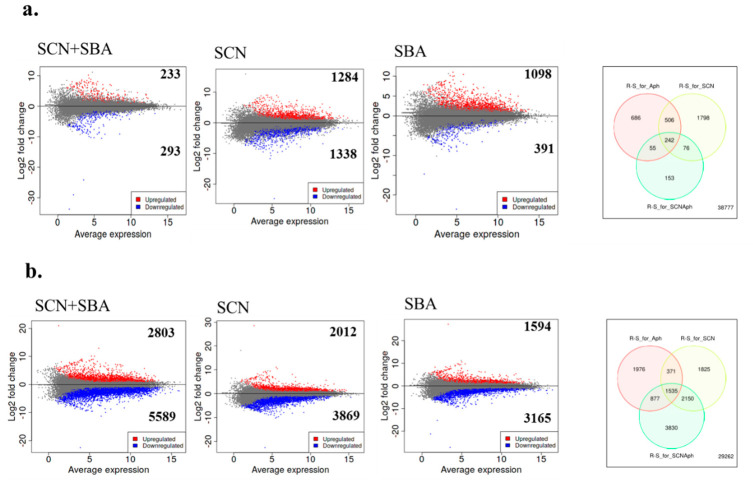
Visualization of DEGs using MA plots and Venn diagrams from resistant-versus-susceptible cultivar contrasts at (**a**) 5 dpi and (**b**) 30 dpi. In MA plots, red = upregulated DEGs, blue = downregulated DEGs, and grey = not significantly differentially expressed genes. In figure labels, R = resistant, S = susceptible, Aph = soybean aphid (SBA), and SCNAph = combined SCN + SBA treatment.

**Figure 4:**
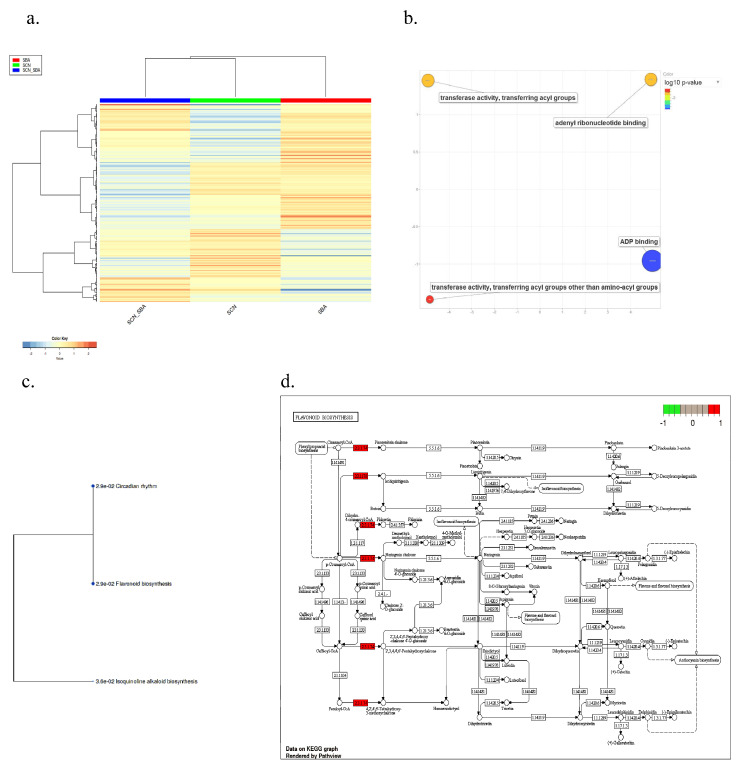
Shared DEGs among SBA, SCN, and SBA + SCN treatments at 5 dpi in resistant-versus-susceptible cultivar contrasts. (**a**) Heatmap of log2 fold-change values. (**b**) Enriched GO molecular functions. (**c**) Hierarchical clustering of enriched KEGG pathways. (**d**) Flavonoid biosynthesis pathway with overrepresented genes. The color scale in panel (**d**) indicates the full range of mapped expression values from −1 to +1, with green representing negative values/down-regulation, white or gray representing little or no change, and red representing positive values/up-regulation. The absence of green pathway nodes indicates that no mapped genes in this pathway panel showed negative values within the displayed scale.

**Figure 5:**
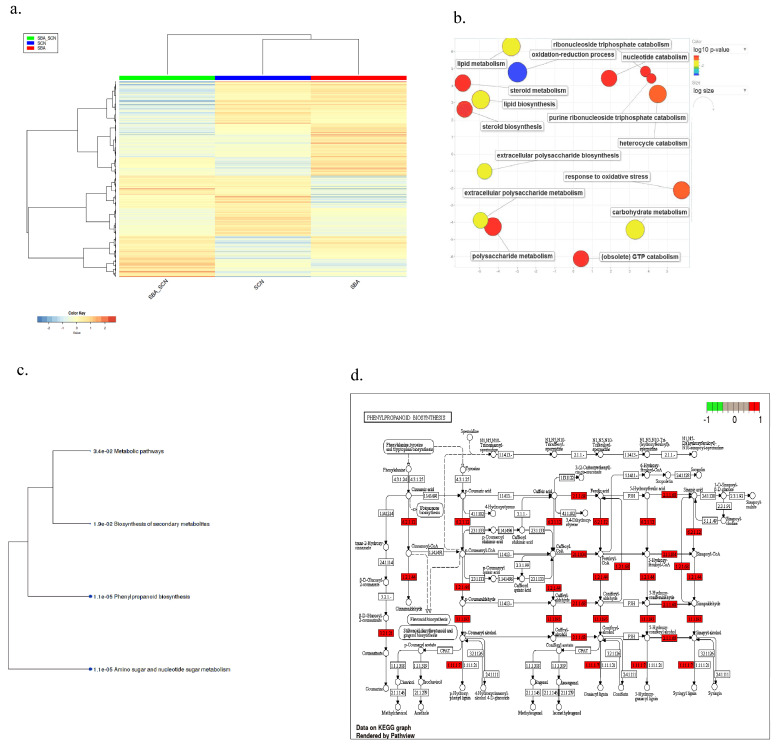
Shared DEGs among SBA, SCN, and SBA + SCN treatments at 30 dpi in resistant-versus-susceptible cultivar contrasts. (**a**) Heatmap of log2 fold-change values. (**b**) Enriched GO biological processes. (**c**) Hierarchical clustering of enriched KEGG pathways. (**d**) Phenylpropanoid biosynthesis pathway with overrepresented genes. The color scale in panel d indicates the full range of mapped expression values from −1 to +1, with green representing negative values/down-regulation, white or gray representing little or no change, and red representing positive val-ues/up-regulation. The absence of green pathway nodes indicates that no mapped genes in this pathway panel showed negative values within the displayed scale.

We compared resistant vs. susceptible cultivars within each treatment at 5 dpi and 30 dpi. Using stringent thresholds (FDR < 0.01; fold change > 2), we identified 4637 differentially expressed genes (DEGs) at 5 dpi and 19,032 DEGs at 30 dpi across SCNs, SBAs, and SCN–SBA contrasts. Despite this extensive divergence, a substantial subset of genes behaved consistently across all three pest contexts within each time point (Figure 3). Notably, 242 DEGs overlapped among all treatments at 5 dpi, and 1535 DEGs overlapped among all treatments at 30 dpi (Figure 3). At 5 dpi, the shared 242-gene set was enriched for molecular functions associated with acyl-transfer chemistry and nucleotide/ADP binding and for pathways including circadian rhythm and flavonoid-related metabolism (Figure 4). Promoter motif enrichment suggested involvement of homeodomain and MYB-related regulatory families. At 30 dpi, the shared 1535-gene set was enriched for oxidation–reduction functions and broad primary/secondary metabolism, including amino sugar/nucleotide sugar metabolism and phenylpropanoid biosynthesis (Figure 5), with promoter motifs implicating AP2, bHLH, bZIP, MYB and additional plant regulatory families. Collectively, these results define (i) an early shared signature with signaling/biosynthetic features and (ii) a later shared signature dominated by redox and metabolic remodeling.

We first focused on the DEGs shared among the SBA, SCN, and combined SBA + SCN treatments, as shown in Figure 4 and Figure 5, to identify core root responses that were consistently different between the resistant and susceptible cultivars under pest pressure. However, genes that respond only to a specific treatment or to the combined SBA + SCN interaction may also provide important biological insight. Therefore, we also examined treatment-specific DEGs, with a particular focus on genes uniquely detected in the resistant cultivar under combined infestation. This analysis identified four resistant-cultivar-unique DEGs at 5 dpi and 100 DEGs at 30 dpi, which may represent candidate genes involved in the soybean response to combined aboveground and belowground pest pressure.

### 2.5. A Subset of DEGs Coincide with Known SCN Resistance QTL Regions (QTL-Coincident Signals)

**Figure 6:**
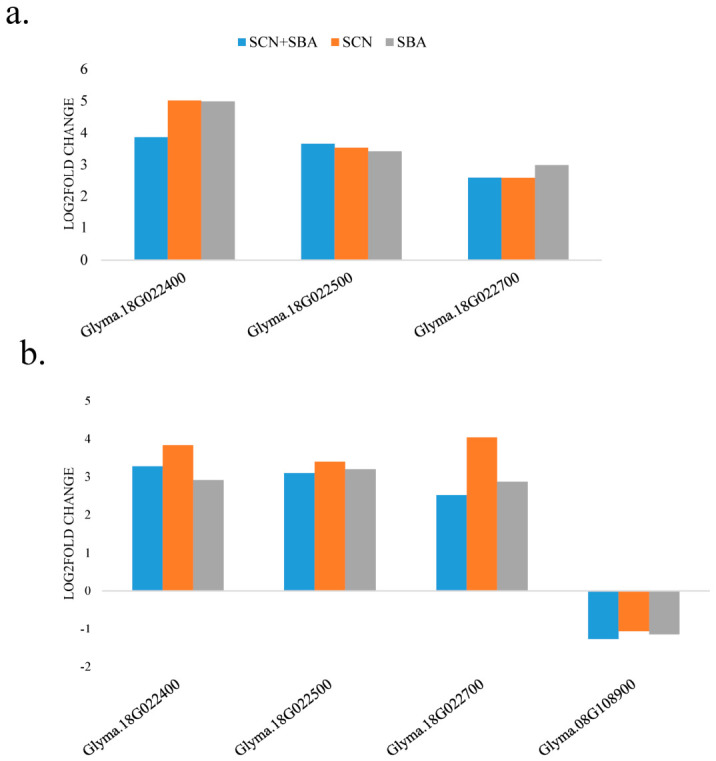
Log2fold change of the DEGs found to be coincident with SCN QTLs upon a comparison of the DEGs between susceptible and resistant cultivars: (**a**) 5dpi and (**b**) 30 dpi.

To connect the transcriptomic results with known soybean resistance regions, we compared the DEGs with soybean genes located within previously reported SCN resistance QTL intervals. This analysis was used to identify differentially expressed soybean genes that co-localized with genomic regions previously associated with SCN resistance rather than genes from the nematode genome. Information on SCN resistance QTLs was obtained from the SoyBase database (https://www.soybase.org (accessed on 16 January 2019)). Thirteen genes were identified from the shared core sets localized to SCN QTL regions: three from the 5 dpi shared set and ten from the 30 dpi shared set (Figure 6). Among the most notable were Glyma.18G022400 (amino-acid-transporter-like), Glyma.18G022500 (GmSNAP18), and Glyma.18G022700 (wound-induced protein), which were consistently upregulated (reported range: ~2.53 to 5.01 log2 fold change) in the resistant cultivar relative to the susceptible cultivar across time points and treatments. These genes co-localize with the rhg1 region associated with durable SCN resistance, supporting consistency between transcriptomic patterns and genetic evidence.

### 2.6. Within-Cultivar Contrasts Show Fewer DEGs in the Resistant Genotype and Identify Interaction-Focused Candidates

We examined treatment effects within each cultivar to identify genes responsive to pest challenges in SCN-resistant (MN1806CN) versus SCN-susceptible (Williams 82) cultivars. Across contrasts, MN1806CN generally exhibited fewer DEGs than the susceptible genotype, consistent with a more buffered transcriptional response. In the resistant cultivar under combined SCN + SBA treatment, only four DEGs were treatment-specific at 5 dpi—Glyma.03G044900 (dirigent-like), Glyma.13G147600 (2OG-Fe(II) oxygenase), Glyma.16G214400 (Exo70B1), and Glyma.20G089400 (proteasome component domain)—constituting a compact, interpretable set consistent with defense signaling/trafficking and proteostasis (Table 1). At 30 dpi, 100 of 19,032 DEGs were uniquely differentially expressed in the resistant cultivar, including Glyma.04G096400 (strong induction, cystatin/cysteine-type endopeptidase inhibitor activity), multiple cytochrome P450s, and a prominent UDP-glucosyltransferase theme, consistent with hormone/JA-related turnover and specialized-metabolite modification (Table 1 and Table 2).

### 2.7. Transcription Factor Motif Enrichment Suggests Distinct Regulatory Regimes in Early vs. Late Responses

Promoter motif enrichment analyses (300 bp upstream) of DEGs indicatted a time-structured regulatory architecture. In MN1806CN under combined infestation, the early response showed enrichment for motifs linked to homeodomain and WRKY-related families among induced genes, whereas late responses showed strong WRKY/TBP/bHLH signatures among repressed genes and TBP/bZIP/MYB/AT-hook/bHLH signatures among induced genes (Table 3). These patterns are consistent with layered defense regulation where early recognition and signaling cascades transition into longer-term metabolic and redox remodeling.

### 2.8. Pathway-Level Analysis Separates Early Lipid/Defense Signaling from Late Carbohydrate and Specialized Metabolism

**Figure 7:**
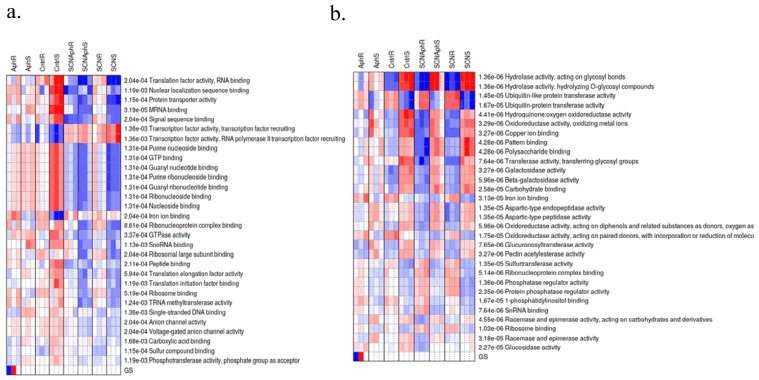
Gene Ontology (GO) molecular annotations overrepresented at **(a)** 5 dpi and **(b)** 30 dpi based on PGSEA analysis. Red and blue indicate higher and lower pathway activities, respectively. In heatmap labels, R = resistant cultivar, S = susceptible cultivar, Aph = soybean aphid (SBA)**,** and SCNAph = combined SCN + SBA treatment.

**Figure 8:**
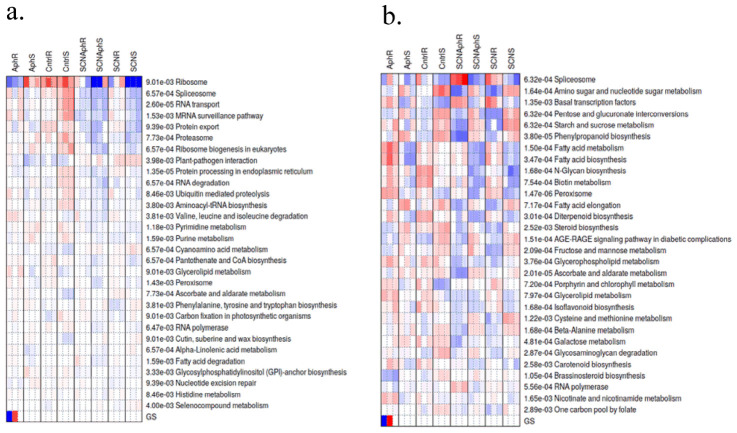
KEGG pathways overrepresented at **(a)** 5 dpi and **(b)** 30 dpi based on PGSEA analysis. Red and blue indicate higher and lower pathway activities, respectively. In heatmap labels, R = resistant cultivar, S = susceptible cultivar, Aph = soybean aphid (SBA), and SCNAph = combined SCN + SBA treatment.

**Figure 9:**
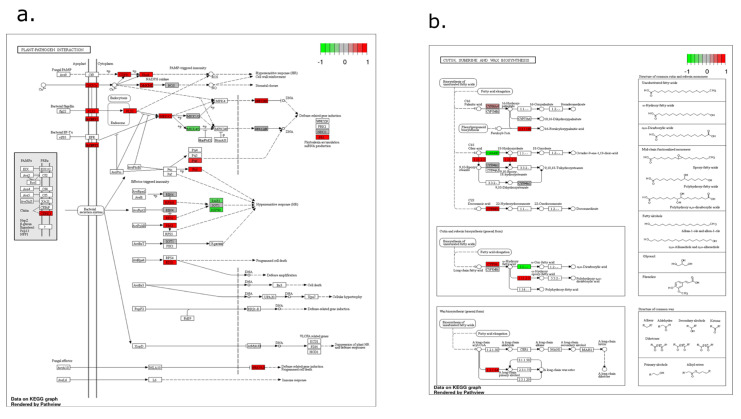
Expression profiles of (**a**) plant–pathogen interaction and (**b**) cutin, suberin, and wax biosynthesis pathways in the resistant cultivar under combined SBA + SCN infestation at 5 dpi. Red and green indicate induced and suppressed genes, respectively.

**Figure 10:**
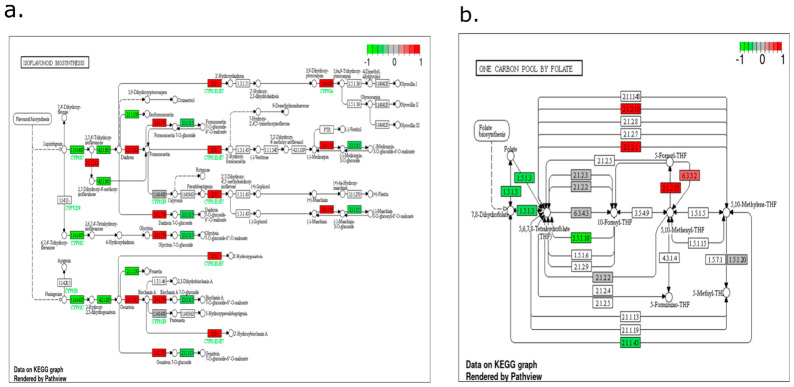
Expression profiles of (**a**) isoflavonoid biosynthesis and (**b**) one-carbon pool by folate pathways in the resistant cultivar under combined SBA + SCN infestation at 30 dpi. Red and green indicate induced and suppressed genes, respectively.

PGSEA/KEGG analyses of the most variable genes showed clear temporal partitioning of pathway activity. At 5 dpi, enriched pathways were dominated by immune signaling and wound-associated lipid processes, including plant–pathogen interaction, ubiquitin-mediated proteolysis, cutin/suberin/wax biosynthesis, α-linolenic acid metabolism, and fatty acid degradation (Figure 7, Figure 8 and Figure 9). By 30 dpi, pathway enrichment shifted toward primary metabolism (multiple carbohydrate pathways), fatty acid biosynthesis/elongation, phenylpropanoid and isoflavonoid biosynthesis, and one-carbon metabolism by folate (Figure 7, Figure 8, Figure 9 and Figure 10). This temporal progression suggests that early defense emphasizes signaling and barrier/wound chemistry, whereas late defense involves extensive resource reallocation and secondary-metabolite deployment.

## 3. Discussion

### 3.1. Host Genotype Shapes the Direction of SCN–SBA Interactions

This study integrated demographic data with root transcriptomics to examine the interactive effects of SCNs and SBAs on soybean using the SCN-resistant cultivar MN1806CN and the SCN-susceptible cultivar Williams 82. Our greenhouse design followed the general approach reported by McCarville et al. [6] but differed in key parameters such as cultivar selection, aphid density, and sampling schedule. At 30 dpi, McCarville et al. [6] observed a 34% increase in SCN eggs and a 28% increase in SCN females on the tested resistant cultivar (DeKalb 27–52, which has PI 88788-derived SCN resistance) in the presence of SBAs. However, the same study observed a decrease in SCN eggs and females on the susceptible cultivar (Kenwood94) in the presence of SBAs at 30 dpi [6]. Interestingly, this study observed a significant SCN egg increase on the susceptible cultivar in the presence of SBAs, but no difference was observed for SCN eggs on the resistant cultivar. It is important to note that although there was not a significant difference for the resistant cultivar, the total SCN egg counts for both treatments on the resistant cultivar increased by approximately 120%. A possible explanation for the difference in results between the two studies is the difference in sources of SCN resistance. The SBA response also differed between soybean genotypes. At 30 dpi, aphid abundance was lower in plants also infested with SCNs, but the magnitude of this reduction varied between the SCN-resistant and SCN-susceptible cultivars. This pattern is consistent with genotype-dependent aboveground–belowground interactions, potentially involving systemic responses linking root defense status with shoot herbivore performance. However, because the present study did not directly test the physiological mechanism underlying aphid suppression, this interpretation should be considered hypothesis-generating.

Previously, Heeren et al. (2012) [21] utilized resistant and susceptible lines with respect to both soybean aphids and SCNs to study the interactive effect of soybean aphids and SCNs under field conditions. Soybean aphid feeding did not affect SCN reproduction in any cultivar, likely due to low SCN egg densities (fewer than 100 eggs per 100 cc of soil) and low aphid populations (fewer than 10 aphids per plant for less than 10 days) observed in some cultivars. We analyzed our data with respect to the aphid counts at 5 dpi, 15 dpi, and 30 dpi to see the trend regarding the SBA populations. At 5 dpi and 15 dpi, we could not observe a significant difference in the SBA counts between all types of treatments. However, at 30 dpi, we observed a significant difference in the SBA counts between all types of treatments. Facilitation at lower herbivore densities and competition at higher herbivore densities might be the reason for differences in the population densities of aphids depending on the length of the experiment [32,33]. Particularly, we observed a 90% decrease in aphid counts on susceptible plants and a 25% decrease in aphid counts on resistant plants exposed to SCNs. The decline in the SBA populations compared to the plants that were not exposed to SCNs might be due to competition for food resources, as both herbivores absorb nutrients via phloem and affect each other [32,34]. A similar pattern was also observed in a study by McCarville et al. (2014) [6]: SBA populations declined at 30 d and 60 d in experiments when infested with five and ten SBAs.

The apparent negative relationship between SCN reproduction and SBA abundance at 30 dpi should be interpreted cautiously. Figure 1b combines both cultivars and treatments, so it is best viewed as a descriptive summary rather than evidence of a treatment-independent mechanism. The treatment-specific results in Figure 1a,c are more informative biologically. In the susceptible cultivar, SBAs increased SCN reproduction, whereas this effect was not observed in the resistant cultivar. In contrast, SCN infestation reduced SBA abundance, but the magnitude of this response differed between cultivars. Together, these results suggest that SBA–SCN interactions are genotype-dependent and that aboveground and belowground pest responses are shaped by the soybean genetic background [35,36,37,38,39,40,41,42,43]. This interpretation is consistent with previous studies showing that belowground herbivores and plant-parasitic nematodes can influence aboveground herbivore performance through systemic changes in host physiology and defense. For example, *Pratylenchus penetrans* infection in *Brassica nigra* reduced infestation by *Pieris rapae* [35]. Nematode infection reduced aphid fertility in grasses, an effect attributed to altered phloem amino acids [36], and reduced offspring performance in *Myzus persicae* on *Plantago lanceolata* infected with *P. penetrans* [37]. Detrimental effects of nematodes on aphids have also been reported in *Brassica* systems [38] and in *Ammophila arenaria* with multiple nematode taxa [40]. Mechanistically, nematode-induced changes in leaf traits (e.g., cuticle wax, leaf toughness, or water content) may reduce aphid suitability [39], and water stress can disproportionately affect phloem-feeding insects [41]. In addition, increased phenolics and related defense chemistry can contribute to reduced shoot herbivory [35,42,43]. Importantly, we did not directly measure the physiological traits that could explain aphid suppression, such as phloem amino acid composition, leaf cuticle thickness, plant water content, or phenolic accumulation. Therefore, these mechanisms should be viewed as possible explanations rather than direct conclusions from this study. Overall, the demographic results indicate a genotype-dependent SBA–SCN interaction and provide biological context for interpreting root-transcriptomic responses under single and combined pest pressure.

### 3.2. Time-Resolved Transcriptomes Reveal an Early Signaling Phase and a Late Metabolic/Redox Phase

We leveraged RNA-seq to characterize root-transcriptional responses to SBA, SCN, and co-infestation at two time points (5 dpi and 30 dpi). RNA-seq provides more sensitive and reproducible transcript measurements across broad dynamic ranges relative to conventional approaches [44]. To reduce analytical complexity and better capture temporal dynamics [45], 5 dpi and 30 dpi datasets were analyzed separately. Across analyses, early responses (5 dpi) were dominated by defense signaling and biosynthetic activities consistent with rapid recognition and response programs, whereas late responses (30 dpi) showed large-scale transcriptional reprogramming involving redox processes, carbohydrate metabolism, and secondary metabolism. The enrichment of hydrogen peroxide and reactive-oxygen-species-related processes specifically at 30 dpi is consistent with a shift toward oxidative stress and redox remodeling during sustained infestation, potentially reflecting longer-term defense activation and altered root physiology under chronic pest pressure.

### 3.3. Candidate Mechanisms for MN1806CN Resilience Under Combined SCN–SBA Pressure

In this study, the identification of DEGs in resistant and susceptible cultivars was a primary objective, particularly under combined SCN and SBA treatments, to examine genes differentially expressed during their interaction. We conducted a comparison between and within the cultivars. Upon comparison, the discordant expression of genes, particularly in the resistant cultivar, was considered important. In total, we found 4 and 100 discordantly expressed DEGs in the resistant cultivar at 5dpi and 30 dpi, respectively. At 5 dpi, Dirigent-like protein, 2OG-Fe (II) oxygenase superfamily), Exo70 exocyst complex subunit, and proteasome component domain protein were differentially expressed in the resistant cultivar that was exposed to both SCNs and SBAs. Dirigent (DIR)-like proteins’ activity is particularly induced in different kinds of biotic (such as wounding) and abiotic stresses, ranging from drought, cold, abscisic acid (ABA), H_2_O_2_, salinity, and osmotic stress [46,47,48]. These proteins play a crucial role in plant defenses against pathogens and lignin and lignan formation [49]. A Dirigent-like protein (Glyma.03G044900) was upregulated by 8.04 log2foldchange at 5 dpi. *Glyma.03G044900*, which encodes a dirigent-like protein, was strongly induced in the resistant cultivar under combined infestation at 5 dpi. Dirigent and dirigent-like proteins are associated with lignin/lignan biosynthesis and plant defense responses to wounding, pathogens, and insect herbivory. This suggests that *Glyma.03G044900* may contribute to early cell-wall or defense-related responses in the resistant cultivar under combined SBA + SCN pressure. DIR-like proteins were also upregulated in spruce (Picea spp.) during feeding by boring insects (i.e., the white pine weevil, *Pissodes strobi*) and by defoliating insects (i.e., the western spruce budworm, *Choristoneura occidentalis*) in bark tissue and green apical shoots [46]. In soybean, *GmDIR22* conferred resistance to *Phytophthora sojae* regulating lignan biosynthesis [50]. Another gene, *Glyma.16g214400*, which belongs to the exocyst subunit exo70 family protein B1, was upregulated by 7.50 log2fold change. The exocyst subunit Exo70B1 interacts with soluble N-ethylmaleimide-sensitive factor attachment protein receptor (SNARE) complex protein SNAP in the process of vesicular trafficking, which mediates the exocytosis [51,52]. *α-SNAP*, which is one of the important genes in *Rhg1*-mediated SCN resistance, has been found to play a role in SCN resistance in many studies [53,54,55,56]. SNAP proteins are involved in vesicle trafficking, which affects the exocytosis of food in syncytia, which in turn affects nematode physiology [53]. Another important DEG is *Glyma.13G147600* (2OG-Fe (II) oxygenase superfamily), which was downregulated by 3.59 log2fold change. The 2OG-Fe(II) oxygenase superfamily, which constitutes flavone synthase I (FNS I), flavonol synthase (FLS), anthocyanidin synthase (ANS), and flavanone 3β-hydroxylase (FHT), plays an important role in flavonoid biosynthesis [57,58]. The remaining DEG, *Glyma.20G089400* (Proteasome component domain protein), was also downregulated by 1.04 log2fold change. Plant proteasomes play important roles in the auxin signaling pathway, oxidative stress, and hypersensitive responses, which are important components of plant defenses [59,60].

At 30 dpi, we found 100 DEGs that were uniquely expressed in the resistant cultivar, of which only 21 genes were upregulated. Plant cystatins can contribute to defense against pests and nematodes by inhibiting proteases involved in feeding or development. The strong induction of *Glyma.04G096400* at 30 dpi may therefore represent a late defense response in the resistant cultivar under combined SBA + SCN infestation. Cystatins are basically low-molecular-weight proteins that inhibit various exogenous proteases or digestive enzymes of invasive pests and pathogens [61,62]. It has been demonstrated that the serine protease activity of *H. glycines* can be inhibited by cowpea trypsin inhibitor (*CpTI*) [63]. Numerous studies on the expression of both native and transgenic cystatins have documented resistance to phytonematodes in a wide range of hosts [64]. The transgenic expression of rice cystatin in eggplant improved resistance against the root knot nematode, *Meloidoyne incognita* [64]. Three DEGs belonged to the cytochrome P450 family [*Glyma.03G160100* (*CYP94B1*), *Glyma.10G115900* (*CYP71B34*), and *Glyma.12G239100* (*CYP712A1*)]. *CYP94* genes play important roles in the Jasmonic acid signaling pathway via catalyzing the sequential ω-oxidation of JA-Ile [65], whereas *CYP71* play a role in flavonoid biosynthesis by producing isoflavone and pterocarpan derivatives such as glyceollin in soybean in pathogen-infected tissues [66]. RNA-seq analysis of two *Glycine soja* genotypes, PI 424093 and PI 468396B, upon infestation by HG type 2.5.7 revealed upregulation of JA, including SA, and the ET pathway [67]. Upon pathway enrichment of 100 DEGs, six genes (*Glyma.01G046300*, *Glyma.09G128300*, *Glyma.09G162400*, *Glyma.11G000500 Glyma.14G175400*, and *Glyma.16G158100*) were enriched for glucosyltransferase activity, and four genes (*Glyma.06G079900*, *Glyma.12G089800*, *Glyma.03G157800*, and *Glyma.13G191200*) were enriched for calcium-binding activity. Previously, the role of glucosyltransferase was shown in Mi-mediated nematode resistance in tomato [68]. This molecule plays an important role in carbohydrate and cell wall biosynthesis [69]. The involvement of calcium/calmodulin-mediated signaling has been shown in responses to *H. glycines* infection in *G. soja* [67].

### 3.4. Expression Patterns of SCN QTL-Linked Genes Support Known Resistance Mechanisms

We examined DEGs that coincided with the 251 genes that were assessed from the SCN QTLs. Remarkably, we found three genes at 5 dpi and ten genes at 30 dpi located in SCN QTLs among common genes in comparisons of resistant versus susceptible cultivars. The main purpose was to determine if the resistant cultivar, MN1806CN, provides *rhg1*- and *Rhg4*-mediated resistance. Three genes—*Glyma.18G022400* (Transmembrane amino acid transporter protein), *Glyma.18G022500* [soluble N-ethylmelaimide sensitive factor (NSF) attachment protein (*GmSNAP18*)], and *Glyma.18G022700* (wound-induced protein WI12)—that were upregulated at both 5 and 30 dpi in all treatments are important for *rhg1*-mediated SCN resistance [53,54,55,70,71]. We did not identify the *Rhg4* gene as a DEG at 5 dpi, and it was downregulated at 30 dpi in all treatments. This indicates that the resistant cultivar, MN1806CN, might possess *rhg1*-mediated SCN resistance [17].

### 3.5. Pathway Enrichment Supports a Working Model Integrating Defense Signaling and Resource Dynamics

To understand the biological functions of the genes, PSGEA analysis was carried out. PSGEA analysis showed distinct enriched pathways at 5 dpi and 30 dpi. Plant–pathogen interaction; ubiquitin-mediated proteolysis; phenylalanine, tyrosine, and tryptophan biosynthesis; cutin, suberin, and wax biosynthesis; alpha-linolenic acid metabolism; and fatty acid degradation pathways were enriched at 5 dpi. Plant–pathogen interaction and ubiquitin-mediated proteolysis play important roles in plant immunity. The interaction between plants and pathogens involves pathogen-associated molecular patterns (PAMPs) of pathogens detected by pattern-recognition receptors (PRRs) of the host and is regulated by E3 ubiquitin ligase [60,72]. E3 ubiquitin ligase has been previously reported to play a role in phytonematodes such as *Heterodera schachtii* [73] and *Globodera rostochiensis* [74]. The phenylalanine, tyrosine, and tryptophan biosynthesis pathways are related to the shikimate pathway. It has been shown that the chorismite mutase enzyme present in root-knot nematodes and potato cyst nematodes alters the shikimate pathway of the host plant [75]. Other pathways such as cutin, suberin, and wax biosynthesis; alpha-linolenic acid metabolism; and fatty acid degradation are related to pathways related to plant lipids, which are important for the production of JA, cutins, and suberins in plant defense via wounding [76,77]. At 30 dpi, most of the pathways were related to carbohydrate metabolism; fatty acid metabolism, including fatty acid biosynthesis and fatty acid elongation; phenylpropanoid biosynthesis; isofalvonoid biosynthesis; and one-carbon pool by folate. The phenylpropanoid biosynthesis and isoflavonoid biosynthesis pathways are particularly related to the type of metabolism that produces various compounds such as flavonoids, anthocynanins, lignin, suberin, salicylic acid, coumarins and furanocoumarins [58,78]. It has been shown that a phloem-feeding insect, namely, the whitefly *Bemisia tabaci*, when infesting *Nicotiana tabacum*, activates the phenylpropanoid pathway [79]. We expected the pathway for carbohydrate metabolism to be enriched at 30 dpi, as SBAs and SCNs might compete for the limited food resources of the plant [33,80]. The pathway one-carbon pool by folate is related to folate-mediated one-carbon metabolism. It has been shown that the *SHMT* (*GmSHMT08*) gene is the resistance gene at the *Rhg4* locus that catalyzes the transformation of the methylene carbon of glycine into tetrahydrofolate (THF) [81,82]. *GmSHMT08* changes its enzymatic properties in the resistant allele that negatively affects folate homeostasis in the syncytium, resulting in hypersensitive responses (HR) leading to programmed cell death (PCD) [27,81,83].

### 3.6. Practical Implications and Limitations

This study shows that soybean genotype influences root-transcriptomic responses to single and combined soybean aphid (SBA) and soybean cyst nematode (SCN) infestation. Comparisons between the SCN-resistant and SCN-susceptible cultivars showed broad differences in gene expression. In contrast, the within-cultivar analysis identified a smaller group of treatment-responsive DEGs in the resistant cultivar under combined SBA + SCN infestation.

Functional enrichment, co-expression, and transcription-factor motif analyses pointed to several defense-related processes. Early responses were mainly associated with signaling and lipid-related pathways, whereas later responses involved redox activity, carbohydrate metabolism, phenylpropanoid and isoflavonoid biosynthesis, and one-carbon metabolism. Several DEGs also overlapped with known SCN resistance QTL regions, suggesting that they may be useful candidate genes for future work. However, these genes should be considered candidate resistance-associated genes, not validated resistance genes.

There are several limitations to this study. First, the results are based mainly on transcriptomic data, so the identified genes, pathways, co-expression modules, and QTL-coincident genes will need functional validation. Second, the SCN-resistant cultivar used in this experiment was still susceptible to SBAs, so we could not evaluate a genotype with resistance to both pests. Third, factors such as pest density, insecticide treatment, drought stress, SBA biotype, SCN HG type, and broader soybean genetic background were not tested here. In addition, RNA-seq was performed using whole-root tissue, which may have masked responses that occur only in specific cell types or infection sites. Overall, these results provide a foundation for understanding how soybean genotype shapes responses to interacting aboveground and belowground pests. Future studies using functional assays, broader soybean germplasms, contrasting SBA biotypes and SCN HG types, field experiments, and infection-site-specific transcriptomics will be important before these candidate genes or loci can be used in marker-assisted or genomic selection for durable pest resistance.

## 4. Materials and Methods

### 4.1. Plant Material and Pest Populations

Two soybean cultivars were used: Williams 82 (PI 518671; SCN-susceptible) and MN1806CN (SCN-resistant to *Heterodera glycines* HG type 0/race 3). Soybean aphid (*Aphis glycines*) biotype 1 colonies were obtained from The Ohio State University and maintained on a susceptible soybean line, LD12-15838R. The SCN population used in this study was HG type 0/race 3, defined by <10% reproduction on indicator lines and avirulence with respect to major SCN resistance sources. The SCN population was maintained and increased on SCN-susceptible soybean plants under greenhouse conditions before use in the experiment.

### 4.2. Greenhouse Experimental Design and Inoculation/Infestation

Experimental design, infestations, sampling, RNA extraction, library preparation, and sequencing are elaborated in the first author’s PhD dissertation work [84] and published as Data Descriptor [31]; key parameters are summarized here. A randomized complete block design was implemented with six blocks for replication. Within each block, a factorial design was implemented, with the first factor being soybean genotype, the second factor being SBA infestation, and the third factor being SCN infestation. For each soybean genotype, treatments included a control (no pests), SBAs only, SCNs only, and SCNs + SBAs. For this experiment, a modified version of the Standard Cyst Evaluation-2008 (SCE-08) protocol was used according to McCarville et al.’s instructions [6,85]. Each block consisted of soybean plants that were planted in 164 cc cone-tainers (3.81 mm diameter; 20.96 mm depth) that were placed in a 7.5 L bucket that was filled with 7 L of construction sand [6]. The sand-filled buckets were maintained in a water bath that was kept at an average temperature of 26.7–28.9 °C to encourage optimal SCN growth. Two soybean seeds for each genotype were planted in the cone-tainers and thinned to one plant per cone-tainer at the V2 growth stage. A 16 h light/8 h dark cycle was used to encourage soybean growth and development. The individual cone-tainers were filled with 125 cc of a 3:1 soil–sand mixture that was prepared using construction sand and clay soil. For SCN treatments, each cone-tainer received 93.75 cc of SCN-infested clay soil that contained approximately 2000 SCN eggs. For SBA treatments, V2 soybean plants were infested with 15 mixed-age biotype-1 SBA using a fine-tip (000) paint brush. The SBAs were applied to the abaxial surface of the first trifoliate. SCN-inoculated soil was used at planting to allow early root infection and subsequent nematode development, whereas SBAs were introduced at the V2 stage because aphid establishment requires sufficient aboveground plant tissue. Thus, the staggered inoculation design reflected the biological requirements of each pest rather than an assumption that both pests interact simultaneously beginning at planting. Buckets were covered with a fine-mesh netting to prevent aphid movement between experimental units.

### 4.3. Aphid Counts and SCN Egg Quantification

Soybean aphid populations were directly/manually counted on each plant at 5, 15, and 30 days post-SBA infestation (dpi). SCN eggs were quantified at 30 dpi. For SCN egg extraction, soil and roots were washed, and the suspension was passed through an 850 µm sieve and retained on a 250 µm sieve. Females/cysts collected on the 250 µm sieve were macerated using a motorized rubber stopper to release eggs; eggs were recovered on a 25 µm sieve after passing through a 75 µm sieve. Eggs were suspended in 50 mL of water and counted manually under a compound microscope using a 1 mL aliquot as a representative sub-sample.

### 4.4. Root Sampling, RNA Extraction, Library Preparation, and Sequencing

Root transcriptomes were analyzed at 5 and 30 dpi to capture early and late stages of the soybean response to pest pressure. The 5 dpi time point was intended to represent early transcriptional responses after SBA establishment and during early SCN infection, whereas 30 dpi represents a later stage when SCN reproduction can be assessed by egg recovery and when aphid population differences among treatments are more evident. Aphid abundance was therefore monitored at 5, 15, and 30 dpi, while SCN egg counts were quantified at 30 dpi. For RNA-seq analysis, whole-root samples from individual plants were used, with three independent biological replicates for each treatment at each time point. The whole-root tissue was collected at 5 and 30 dpi by snap-freezing it in liquid nitrogen and storing it at −80 °C. Frozen roots were ground into a fine powder in liquid nitrogen, and total RNA was extracted using the PureLink RNA Mini Kit (Invitrogen, Carlsbad, CA, USA). RNA was DNase-treated (TURBO DNase, Invitrogen), integrity was assessed by 1% agarose gel electrophoresis, and concentration was measured using a NanoDrop 2000 (Thermo Fisher Scientific, Waltham, MA, USA). cDNA libraries were prepared with the NEBNext Ultra II RNA Library Prep Kit (96 single index) and sequenced on an Illumina HiSeq 3000 platform (single-end, 100 bp) at the Iowa State University Sequencing Facility.

### 4.5. RNA-Seq Preprocessing, Mapping, Quantification, and Normalization

Raw read quality was assessed using FastQC (v0.11.3) and summarized with MultiQC (v1.3). Low-quality bases were removed with Btrim64 (v0.2.0) (quality threshold >20; 5-bp sliding window). High-quality reads were quantified by mapping them to soybean primary coding sequences (Wm82.a2.v1; Phytozome) using Salmon (v0.9.1). Downstream analyses were conducted in iDEP (v0.81). Genes were filtered at ≥0.5 counts per million (CPM) in at least one sample, normalized using DESeq2, and transformed using DESeq2’s regularized log (rlog) for clustering/ordination.

### 4.6. Differential Expression and Candidate-Gene Analyses

Raw *p*-values were adjusted for multiple testing using the Benjamini–Hochberg procedure to control the false-discovery rate (FDR) [86]. Differentially expressed genes (DEGs) were identified using DESeq2 [87] with an absolute fold change (|FC|) ≥ 2 and FDR ≤ 0.01. Effects of cultivar, treatment, and their interaction were tested using the following model: expression ~ cultivar + treatment + cultivar: treatment. DEG annotations were obtained from SoyBase, the USDA-ARS soybean genetics and genomics database (https://www.soybase.org/ (accessed on 16 January 2019)).

### 4.7. Co-Expression Networks, Functional Enrichment, and Motif Analysis

To reduce analytical complexity, count matrices were analyzed by time point for network analyses. Weighted gene co-expression networks were constructed in WGCNA using the top 2000 most variable genes, with a soft-threshold power = 5 and minimum module size = 20. Functional enrichment of DEGs (GO terms and KEGG pathways) was performed using ShinyGO, REVIGO, and iDEP. Promoter motif enrichment was assessed by scanning 300 bp upstream regions of DEGs for transcription factor binding motifs (ShinyGO and iDEP). Pathway-level patterns were visualized using MapMan, with functional binning assigned via Mercator. PGSEA was used for gene set enrichment analyses with an FDR-based significance cutoff.

### 4.8. Statistical Analyses for Phenotypes

Aphid and SCN counts were analyzed in GraphPad Prism v8.0.2. SCN egg counts at 30 dpi were analyzed by one-way ANOVA followed by Tukey’s multiple-comparisons test. SBA population counts were analyzed across 5, 15, and 30 dpi using two-way ANOVA with the Geisser–Greenhouse correction, followed by Tukey’s multiple comparisons within each sampling time point. For Figure 1, asterisks indicate significant differences from the indicated post hoc multiple-comparison tests rather than separate unadjusted *t*-tests (ns, not significant; *, *p* ≤ 0.05; **, *p* ≤ 0.01; ***, *p* ≤ 0.001; ****, *p* ≤ 0.0001). Linear regression was used only as a descriptive analysis to evaluate the overall relationship between SBA density and SCN egg counts at 30 dpi. All statistical analyses were conducted using GraphPad Prism version 8.0.2 (GraphPad Software, San Diego, CA, USA).

## Figures and Tables

**Table 1:** Four resistant-cultivar-specific DEGs detected under combined SBA + SCN treatment at 5 dpi.

Gene ID	log2foldchange	*p*-Value	Top Arabidopsis Hit	Gene Description	Gene Ontology Biological Process
Glyma.03g044900	8.04	7.16 × 10^−3^	AT5G49040.1	Disease resistance-responsive (dirigent-like protein) family protein	GO:0006952, GO:0009807
Glyma.13g147600	−3.59	6.27 × 10^−3^	AT2G36690.1	2-oxoglutarate (2OG) and Fe(II)-dependent oxygenase superfamily protein	GO:0009058, GO:0055114
Glyma.16g214400	7.50	4.90 × 10^−3^	AT5G58430.1; ATEXO70B1, EXO70B1	exocyst subunit exo70 family protein B1	GO:0006887, GO:0006904, GO:0009738, GO:0035556
Glyma.20g089400	−1.04	2.81 × 10^−4^	AT5G15610.2	Proteasome component (PCI) domain protein	GO:0006302, GO:0006312, GO:0007062, GO:0007129, GO:0007131, GO:0008150, GO:0009560, GO:0009909, GO:0034968, GO:0042138, GO:0045132

**Table 2:** Enriched functional categories among 100 resistant-cultivar-specific DEGs detected under combined SBA + SCN treatment at 30 dpi.

Enrichment FDR	Genes in List	Total Genes	Functional Category	Genes
0.000104666	6	236	Quercetin 3-O-glucosyltransferase activity	Glyma.01G046300, Glyma.09G128300, Glyma.09G162400, Glyma.11G000500, Glyma.14G175400, Glyma.16G158100
0.000104666	6	236	Quercetin 7-O-glucosyltransferase activity	Glyma.01G046300, Glyma.09G128300, Glyma.09G162400 Glyma.11G000500, Glyma.14G175400, Glyma.16G158100
0.000894105	6	371	UDP-glucosyltransferase activity	Glyma.01G046300, Glyma.09G128300, Glyma.09G162400 Glyma.11G000500, Glyma.14G175400, Glyma.16G158100
0.002120752	6	458	Glucosyltransferase activity	Glyma.01G046300, Glyma.09G128300, Glyma.09G162400 Glyma.11G000500, Glyma.14G175400, Glyma.16G158100
0.004252586	6	544	UDP-glycosyltransferase activity	Glyma.01G046300, Glyma.09G128300, Glyma.09G162400 Glyma.11G000500, Glyma.14G175400, Glyma.16G158100
0.027351491	6	812	Transferase activity, transferring hexosyl groups	Glyma.01G046300, Glyma.09G128300, Glyma.09G162400 Glyma.11G000500, Glyma.14G175400, Glyma.16G158100
0.029538121	7	1139	Transferase activity, transferring glycosyl groups	Glyma.01G046300, Glyma.09G128300, Glyma.09G162400 Glyma.11G000500, Glyma.14G175400, Glyma.16G158100, Glyma.20G004900
0.037769863	2	63	Protein-disulfide reductase activity	Glyma.08G295600, Glyma.18G127400
0.049127578	2	78	Thioredoxin-disulfide reductase activity	Glyma.08G295600, Glyma.18G127400
0.049127578	2	81	Oxidoreductase activity, acting on a sulfur group of donors, with disulfide as an acceptor	Glyma.08G295600, Glyma.18G127400
0.049282369	4	478	Calcium ion binding	Glyma.06G079900, Glyma.12G089800, Glyma.03G157800, Glyma.13G191200

**Table 3:** Enriched transcription factor (TF) binding motifs of DEGs in the resistant cultivar under combined SBA + SCN infestation at 5 and 30 dpi. At 5 dpi, enriched motifs were mainly associated with homeodomain and Myb/SANT families. At 30 dpi, enriched motifs broadened to include AP2, B3, bHLH, bZIP, Myb/SANT, SBP, TCP, and WRKY families.

TF	Motif	TF Family	FDR	TF	Motif	TF Family	FDR
5 dpi (upregulated)				30 dpi (upregulated)			
Glyma0041s00360.1	GCTGTCA	Homeodomain	2.50 × 10^−2^	Glyma03g04500.1	ATA	TBP	1.50 × 10^−8^
Glyma01g42410.1	GTCA	Homeodomain	2.50 × 10^−2^	Glyma08g08220.1	ACACGTG	bZIP	2.80 × 10^−6^
Glyma01g03450.1	GTCA	Homeodomain	2.50 × 10^−2^	Glyma19g30680.1	GACGTG	bZIP	4.30 × 10^−6^
Glyma03g39040.1	TGACGGC	Homeodomain	2.50 × 10^−2^	Glyma01g01740.1	ACGTGG	bZIP	4.30 × 10^−6^
Glyma01g43420.1	GTCAAC	WRKY	3.00 × 10^−2^	Glyma01g00600.1	GGATAA	Myb/SANT	1.30 × 10^−5^
Glyma01g43130.1	GTCAA	WRKY	3.00 × 10^−2^	Glyma01g38380.1	ACGTGGC	bZIP	1.30 × 10^−5^
Glyma07g36640.1	GTCAA	WRKY	3.10 × 10^−2^	Glyma13g43120.1	GGATAA	Myb/SANT	1.30 × 10^−5^
Glyma15g37120.1	GTCAA	WRKY	3.20 × 10^−2^	Glyma04g04170.1	ACACGTG	bZIP	1.30 × 10^−5^
Glyma02g45530.1	GTCAA	WRKY	3.20 × 10^−2^	Glyma06g01700.1	ATATAATT	AT hook	1.90 × 10^−5^
Glyma10g13720.1	GGTCAA	WRKY	3.20 × 10^−2^	Glyma09g06770.1	CACGTGT	bHLH	1.90 × 10^−5^
Glyma03g37870.1	GTCAAC	WRKY	3.20 × 10^−2^	Glyma02g00980.1	CACGTG	bHLH	1.90 × 10^−5^
Glyma02g15920.1	GTCAAC	WRKY	3.20 × 10^−2^	Glyma01g04610.1	CACGTG	bHLH	2.60 × 10^−5^
Glyma01g39600.1	GTCAAC	WRKY	3.80 × 10^−2^	Glyma01g39450.1	CCACGTG	bHLH	4.30 × 10^−5^
Glyma07g05660.1	TTACGTAA	NAC/NAM	3.80 × 10^−2^	Glyma01g39360.1	CACGTG	bHLH	4.80 × 10^−5^
Glyma01g00510.1	TGTCGG	B3	4.40 × 10^−2^	Glyma08g41620.1	GCCACGTG	bHLH	4.80 × 10^−5^
Glyma09g06980.1	GTCAAC	WRKY	5.60 × 10^−2^	Glyma06g41620.1	CACGTG	bHLH	6.20 × 10^−5^
Glyma01g06150.1	GTCAA	NAC/NAM	5.60 × 10^−2^	Glyma01g02250.1	CACGTG	bHLH	6.50 × 10^−5^
Glyma01g21020.1	TGACGTCA	bZIP	5.60 × 10^−2^	Glyma03g04500.1	GGAT	Myb/SANT	6.50 × 10^−5^
Glyma06g17690.1	GTCAAC	WRKY	6.00 × 10^−2^	Glyma08g08220.1	CACGTG	bHLH	8.80 × 10^−5^
Glyma04g06470.1	GTCAA	WRKY	6.70 × 10^−2^	Glyma19g30680.1	CGCGT	CG-1	8.80 × 10^−5^
				Glyma05g36290.1	ATA	TBP	1.50 × 10^−8^
				Glyma03g32740.1	ACACGTG	bZIP	2.80 × 10^−6^
				Glyma05g31190.1	GACGTG	bZIP	4.30 × 10^−6^
				30 dpi (down regulated)			
				Glyma09g06980.1	GTCAAC	WRKY	6.30 × 10^−8^
				Glyma04g08060.1	GTCAA	WRKY	6.30 × 10^−8^
				Glyma01g06870.1	GTCAAC	WRKY	6.30 × 10^−8^
				Glyma03g04500.1	ATA	TBP	1.20 × 10^−7^
				Glyma02g15920.1	GTCAAC	WRKY	1.20 × 10^−7^
				Glyma01g43420.1	GTCAAC	WRKY	2.00 × 10^−7^
				Glyma09g39040.1	GTCAA	WRKY	2.00 × 10^−7^
				Glyma01g09010.1	CACGTG	bHLH	2.60 × 10^−7^
				Glyma03g37870.1	GTCAAC	WRKY	2.60 × 10^−7^
				Glyma19g30680.1	GACGTG	bZIP	3.00 × 10^−7^
				Glyma05g07490.1	CACGTG	bHLH	3.00 × 10^−7^
				Glyma01g43130.1	GTCAA	WRKY	3.10 × 10^−7^
				Glyma02g01420.1	AGTCAACG	WRKY	3.20 × 10^−7^
				Glyma09g39000.1	AGTCAA	WRKY	3.20 × 10^−7^
				Glyma01g39600.1	GTCAAC	WRKY	3.70 × 10^−7^
				Glyma03g32740.1	CACGTG	bHLH	4.50 × 10^−7^
				Glyma02g45530.1	GTCAA	WRKY	4.50 × 10^−7^
				Glyma02g00980.1	CACGTG	bHLH	4.50 × 10^−7^
				Glyma01g05050.1	GTCAACG	WRKY	4.70 × 10^−7^
				Glyma06g17690.1	GTCAAC	WRKY	4.80 × 10^−7^

## Data Availability

All raw sequencing reads have been archived in the NCBI Sequence Read Archive (SRA) under accession range SRR8427366–SRR8427408, associated with BioProject PRJNA514200 (SRA study/Project ID: SRP178193). Untransformed transcript abundance values for every sample are available through the NCBI Gene Expression Omnibus (GEO) under GSE125103. In addition, the processed/transformed transcript abundance matrix, along with the hierarchical clustering outputs, correlation matrices, and cluster assignments, are publicly hosted on Figshare (https://doi.org/10.6084/m9.figshare.7755152.v3).
